# 3D object detection for vehicle-mounted LiDAR based on deep learning and euclidean clustering algorithm

**DOI:** 10.1371/journal.pone.0348581

**Published:** 2026-06-01

**Authors:** Nan Zhang, Maolong Xi, Juan Fang, Fangqin Wang

**Affiliations:** School of Control Engineering, Wuxi University of Technology, Wuxi, China; University of Verona: Universita degli Studi di Verona, ITALY

## Abstract

Object Detection (OD) stands as a fundamental task in the area of autonomous driving environment perception. This study introduces a 3D OD method grounded in deep learning and an improved Euclidean clustering algorithm, aiming to improve the accuracy and efficiency of point cloud segmentation and OD. The core methodological innovations include: (1) the integration of the Cloth Simulation Filter (CSF) for accurate ground and non-ground point separation, combined with a K-Dimensional Tree (KD-Tree) structure and an adaptive parameter mechanism to enhance clustering robustness and efficiency; and (2) an enhanced PointNet architecture incorporating multi-scale grouping (MSG), multi-resolution grouping (MRG), and skip connections to improve local feature extraction and multi-level feature fusion. This method is differentiated from prior works by its holistic integration of density-aware segmentation and hierarchical feature aggregation, addressing key bottlenecks in handling sparse and uneven LiDAR data. The proposed method is rigorously evaluated on the KITTI and NuScenes benchmarks. It achieves segmentation accuracies of 94.96% and 93.12%, with single-frame processing times of 15.63 ms and 17.24 ms, respectively, demonstrating a superior balance of speed and precision compared to traditional Euclidean clustering and other baseline methods. For the 3D OD task, the model attains average detection accuracies of 94.36% and 92.68% on the respective datasets, representing statistically significant improvements (*p* < 0.001) over the standard PointNet. The detection speed reaches 34 fps and 31 fps, meeting real-time requirements while outperforming existing frameworks in challenging scenarios involving occluded and multi-scale objects. The findings confirm that the proposed framework provides a robust, efficient, and generalizable solution for 3D environmental perception in autonomous driving systems.

## 1. Introduction

With the rapid development of smart transportation and self-driving automotive technologies, precise perception of the surrounding environment by vehicles has become a key element in achieving safe and reliable Autonomous Driving (AD), which refers to the capability of a vehicle to navigate and operate without human intervention through the integration of sensors, algorithms, and control systems [1]. As a key sensor in the vehicle perception system, vehicular LiDAR is widely used in Three-Dimensional (3D) OD tasks owing to its capability of producing detailed and precise 3D Point Cloud (PC) data depicting the surrounding environment [2]. However, conventional vehicular LiDAR-based 3D OD approaches mainly rely on manual feature extraction and traditional machine learning techniques. When faced with complex and ever-changing driving scenarios and large-scale PC data, these methods encounter issues such as difficulty in feature extraction, poor generalization ability, and low detection accuracy and efficiency [3]. Therefore, exploring an efficient and high-precision method for 3D OD using vehicle-mounted LiDAR has become a current research hotspot [4].

In recent years, Deep Learning (DL) technology has achieved great success in fields such as computer vision, pedestrian detection, and AD. Its powerful feature learning and automatic extraction capabilities provide a novel solution for solving the problem of vehicular LiDAR 3D OD. DL models are extensively applied in the field of AD due to their capability to automatically learn effective feature representations from large volumes of labeled data, thereby enabling more accurate and efficient 3D OD [5]. For instance, to address the inherent lack of 3D information in monocular images, S. Y. Alaba and J. E. Ball proposed a DL-based method that restores scene geometry through depth estimation and 3D bounding box encoding, demonstrating improved localization and pose estimation accuracy. On the benchmark, for the 3D detection task of the car category under the moderate difficulty level, this method achieved 88.64% average precision [6]. Beyond camera-based approaches, the fusion of LiDAR with other sensors has been explored to enhance robustness. D. Wu et al. surveyed DL methods for LiDAR-only and LiDAR-fusion 3D perception, highlighting the need to balance accuracy with computational complexity, especially when handling adverse weather conditions and multi-source data redundancy. The fusion model with the best performance achieved over 85% mAP on the dataset, but the latency also increased to 200 milliseconds accordingly [7]. Addressing the challenge of effective cross-modality fusion due to significant sensory differences, C. Lin et al. introduced CL3D, a camera-LiDAR 3D detection framework that enhanced geometric features via a point enhancement module and achieved alignment through point-guided fusion [8]. Furthermore, for dynamic tracking, C. Nie et al. developed a LiDAR-camera fusion method employing an interactive multi-model unscented Kalman filter and particle swarm optimization for real-time 3D OD and tracking in highway scenarios [9].

Concurrently, clustering algorithms, as classic unsupervised learning methods, have been widely investigated for organizing unlabeled PC data. To overcome the limitations of real-time performance and hardware deployability in software-based clustering, X. Zhang and X. Huang designed an efficient pipeline hardware architecture implemented on a Field-Programmable Gate Array, achieving ultra-fast single-frame PC processing [10]. Moving towards more semantically aware segmentation, R. Marcuzzi et al. proposed a mask-based panoptic LiDAR segmentation method that utilizes semantic information for clustering, outperforming traditional geometry-only methods. Experiments on the dataset show that the MaskPLS method achieved 71.5% panoptic quality [11]. For specific applications such as roadside perception, J. Wu et al. introduced a real-time clustering algorithm that effectively filters background points and clustering targets based on voxel grid features, with a processing time of only 100ms [12]. In order to improve accuracy and efficiency in the automotive environment, W. Yang et al. developed a clustering method based on 3D grids and an improved density algorithm, which improved detection accuracy by 7.6% and reduced computation time by 16.2% compared to traditional clustering methods [13]. In response to the challenge of unstructured PCs, P.S. Singh et al. proposed a hybrid method that combines random sample consensus algorithm and Euclidean clustering (EC) for hierarchical segmentation of unmanned aerial vehicle PCs, which can accurately segment 91% of point clouds [14]. While these advancements in unsupervised clustering and the development of powerful DL-based detectors have improved handling of occluded shapes and sparse data, a persistent and critical challenge for vehicular applications remains: achieving an optimal balance between high detection accuracy and computational efficiency to meet stringent real-time requirements [15,16].

In addition, current research focuses on domain adaptation and generalization challenges in LiDAR perception. Scholars have proposed a variety of solutions for performance degradation caused by different sensor models, weather and geographical environment. For example, domain adaptation techniques have been proposed to alleviate performance degradation caused by changes in sensor types, weather conditions, and geographic locations. These tasks typically involve adversarial training, self supervised learning on the target domain, or designing domain invariant feature representations [17]. Another important exploration direction involves model efficiency and deployment. Although accuracy is crucial, the auto drive system in the real world needs a solution with high computing efficiency and suitable for embedded hardware [18]. Therefore, a large amount of research has been devoted to developing lightweight network architectures, model compression techniques, and hardware aware neural network designs to achieve real-time inference on resource constrained platforms [19].

In summary, despite extensive research on PC processing methods and 3D OD for vehicle-mounted LiDAR by numerous scholars, significant achievements have been made. However, current methods still exhibit deficiencies such as insufficient robustness in PC segmentation under complex environments, limited ability to extract local features, and poor real-time detection performance. In view of this, a 3D OD method based on an improved EC algorithm and PointNet is proposed. This method enhances the precise segmentation of vehicle-mounted LiDAR PCs by improving the EC algorithm, and utilizes an improved PointNet to better capture local features, achieving efficient and high-precision 3D OD.

The core novelty of this research resides in two key aspects: (1) At the PC preprocessing and segmentation level, a dynamic density-aware hierarchical clustering paradigm is introduced. This method overcomes the limitation of fixed distance thresholds in traditional EC by integrating local PC distribution statistics with global topological constraints, achieving adaptive segmentation of unstructured PCs. Specifically, the study designed a curvature-consistency-based region growing mechanism that effectively suppresses noise and outliers while preserving weakly-featured objects, thereby providing more discriminative candidate regions for subsequent detection tasks. (2) At the 3D OD network design level, a multi-granularity feature co-enhancement detection architecture is constructed. While retaining PointNet’s point-wise feature learning capability, this architecture introduces an attention-guided local structure encoding module, which enhances robustness in occluded and sparse scenes by explicitly modeling geometric relationships between points and their k-nearest neighbors. Furthermore, the study designed a bidirectional feature pyramid fusion path that enables complementary enhancement from shallow fine-grained details to deep semantic information, significantly improving the classification and localization accuracy of multi-scale objects.

The contribution of this study lies in: (1) The novel integration and parameterization of CSF ground filtering, the dynamically adjusted distance threshold based on neighborhood statistics, and the logarithmic point count adaptation, which together form a robust and efficient segmentation pipeline tailored for vehicular LiDAR PCs under varying density and noise levels. (2) The integrated architecture that combines random point sampling for efficiency and a tailored combination of MSG and MRG strategies for robust multi-scale. (3) Feature extraction in driving scenes, and a strategically placed skip connection mechanism to enhance feature fusion.

The remainder of this paper is organized as follows. The Methods and materials section details the proposed methodology, including the improved EC algorithm for PC segmentation and the enhanced PointNet for 3D OD. The Results section presents the experimental setup, results, and comparative analysis on two public datasets. The Discussion section provides an in-depth discussion of the results, highlighting the advantages and limitations of the proposed approach. Finally, The Conclusion section concludes the paper and suggests directions for future research.

## 2. Methods and materials

A method for segmenting vehicle-mounted LiDAR PCs based on an improved EC algorithm is proposed, aiming to enhance the accuracy and efficiency of vehicle-mounted LiDAR PC processing and OD. Subsequently, a 3D OD method based on an improved PointNet is introduced, which achieves high-precision and high-efficiency object recognition and localization through multi-scale feature extraction and skip connection mechanisms.

### 2.1 Vehicle-mounted LiDAR PC segmentation method based on improved EC algorithm

The PC data obtained from vehicle-mounted LiDAR possesses advantages such as high precision and high resolution, but it also faces issues like excessive noise, uneven density, and significant ground interference, directly impacting the accuracy and efficiency of subsequent target detection. Although traditional EC algorithms are widely applied in PC segmentation, they exhibit limitations when dealing with large-scale, high-noise PCs, such as low computational efficiency, sensitivity to parameters, and susceptibility to ground point interference [20]. Therefore, this study proposes a vehicle-mounted LiDAR PC segmentation method based on an improved EC algorithm. By introducing a ground point filtering strategy and an efficient neighbor search mechanism, the robustness and real-time performance of PC segmentation are enhanced. The robustness of PC segmentation is defined as the performance retention rate of the model under changes in PC density and noise level; Real time is defined as the single frame processing time satisfying the sensor frame period constraint. Its expression is shown in Eq (1).


$$\begin{array}{c} \left\{ \begin{array}{c} @l@R=1-\frac{\left|P\left(\rho ,\sigma \right)-P_{\text{base}}\right|}{P_{\text{base}}}\\ T_{\text{proc}}\leq T_{max} \end{array}\right. \end{array}$$ (1)

In Eq (1), R represents robustness. ρ and σ represent PC density and noise level, respectively. $P_{\text{base}}$ represents the benchmark performance indicator. $T_{\text{proc}}$ represents the time required for the system to process a single frame PC. $T_{max}$ represents the upper limit of the sensor frame period. The CSF algorithm divides PCs into ground points and non-ground points by simulating the interaction between cloth and the ground. This algorithm treats the PC as a series of cloth particles, with each particle subjected to the combined effects of gravity and ground reaction force. By iteratively calculating the position changes of each particle, the final stable height of the particles corresponds to the simulated ground height, thus achieving precise filtering of ground points [21]. The force model and position update of cloth particles are presented in Eq (2).


$$\begin{array}{c} \left\{ \begin{array}{c} @l@F_{\text{ext}}=G+R\\ P_{g}(t+1)=2P_{g}\left(t\right)-P_{g}(t-1)+\frac{F_{\text{ext}}}{m}\Delta t^{\text{2}} \end{array}\right. \end{array}$$ (2)

In Eq (2), $F_{\text{ext}}$ is the external resultant force; G and R are gravity and ground reaction respectively; $P_{g}(t+1)$ is the 3D position of particle g at the next time t+1; $P_{g}\left(t\right)$ is the 3D position of particle g at the current time t; m and Δt are particle mass and time step, respectively; $\frac{F_{\text{ext}}}{m}$ is the acceleration of the particle. After the cloth simulation is stable, the study compares the height of each point in the original PC with the height of the cloth particle at the corresponding position, and calculates its absolute height difference, as shown in Eq (3).


$$\begin{array}{c} H_{\text{diff}}=\left|Z_{\text{point}}-Z_{\text{cloth}}\right| \end{array}$$ (3)

In Eq (3), $H_{\text{diff}}$ is the height difference; $Z_{\text{point}}$ is the elevation value of the midpoint of the original PC; $Z_{\text{cloth}}$ is the simulated ground height of the cloth particles at the corresponding position. To improve the nearest neighbor (NN) search efficiency in European clustering, the study constructed a KD-Tree structure to divide the PC space. The KD-Tree is a specialized binary tree structure tailored for arranging points in a k-dimensional space. It recursively partitions the space into two distinct sub-spaces by selecting different dimensions as the segmentation axis and taking the median point on the dimension as the segmentation hyperplane [22]. The node division is presented in Eq (4).


$$\begin{array}{c} Split\left(D,d\right)=\text{median}\left\{P_{d}|p\in D\right\} \end{array}$$ (4)

**Fig 1:**
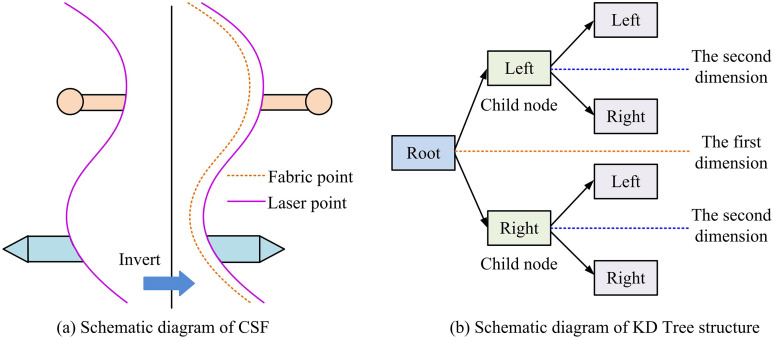
Diagrammatic representation of CSF and KD-Tree structures.

In Eq (4), Split(D,d) is the value of the split point; D is the point set of nodes; d indicates partition dimension; p indicates the point in the point set; $P_{d}$ is the coordinate value of the point on dimension d; median is the median function. CSF is a terrain-following algorithm that simulates the physical interaction between a piece of cloth and the ground surface. By treating the PC as a set of cloth nodes influenced by gravity and internal forces, CSF iteratively fits a cloth mesh to the terrain, effectively separating ground points from non-ground objects, thereby providing a cleaner input for subsequent clustering and detection stages. The schematic diagram of CSF and KD-Tree structure is presented in Fig 1.

Fig 1 illustrates the principle of the key preprocessing techniques used in this study. Fig 1(a) is a schematic diagram of CSF, which simulates the process of fabric particles adhering to ground laser points under the action of gravity, achieving precise filtering of ground points. Fig 1(b) is a schematic diagram of the KD tree structure, which shows the organization of PC data by recursively dividing space according to different coordinate axes, forming a binary tree to accelerate nearest neighbor search. The root node, child nodes, and left and right branches clearly reflect the spatial segmentation logic. When conducting neighbor search based on the KD-Tree structure, a fast and accurate method is required to calculate the distance between two points to determine whether they belong to the same cluster. The KD-Tree structure can quickly retrieve all candidate points near a given point, avoiding global traversal and improving clustering efficiency. The calculation of the NN search distance is presented in Eq (5) [23].


$$\begin{array}{c} d\left(p,q\right)=\sqrt{(x_{p}-x_{q}{)}^{\text{2}}+(y_{p}-y_{q}{)}^{\text{2}}+(z_{p}-z_{q}{)}^{\text{2}}} \end{array}$$ (5)

In Eq (5), d(p,q) is the Euclidean distance between points p and q; p and q denote points in 3D space; $x_{p}$, $y_{p}$ and $z_{p}$ are the 3D coordinates of point p; $x_{q}$, $y_{q}$ and $z_{q}$ are the 3D coordinates of point q. On the basis of traditional EC, adaptive distance threshold and minimum number of points are introduced [24]. To adapt to the change of PC density in different scenes, an adaptive distance threshold calculation method is proposed. This method is based on the average NN distance of the PC to dynamically adjust the clustering radius, as shown in Eq (6).


$$\begin{array}{c} \varepsilon =\alpha \cdot \frac{\text{1}}{N}\sum_{n=1}^{N}d\left(p_{u},p_{u,nn}\right) \end{array}$$ (6)

In Eq (6), ε is adaptive distance threshold; α is the adjustment coefficient; N is the number of non ground PCs; $p_{u,nn}$ is the NN of point $p_{u}$; $d\left(p_{n},p_{n,m}\right)$ is the distance of the NN between two points. α adjusts the distance threshold based on local point density; Larger α allows merging of sparser regions, while smaller α preserves fine structure. In addition, the minimum number of points threshold also needs to be adjusted adaptively according to the size of the PC [25]. In this study, logarithmic function is used to balance clustering sensitivity and computational efficiency, as shown in Eq (7).


$$\begin{array}{c} MinPts=\beta \cdot log\left(N\right) \end{array}$$ (7)

In Eq (7), MinPts is the minimum number of points threshold; β is the adjustment coefficient; log(N) is the logarithm of the number of PCs. β performs logarithmic scaling on the minimum point threshold to prevent over segmentation in dense PCs and under segmentation in sparse PCs. Eq (7) constructs an efficiency model through the logarithmic point number adaptive mechanism MinPts, transforming the control of computational complexity into a scaling problem of logarithmic functions. Its parameter β directly balances the risks of over segmentation and under segmentation. In the process of clustering, it is essential to clearly define the judgment criteria of whether two points belong to the same cluster. This standard comprehensively considers the distance constraint and density requirements to ensure the rationality of the clustering outcomes. The clustering merging conditions are presented in Eq (8).


$$\begin{array}{c} \left\{ \begin{array}{c} @l@\text{if }d\left(p,q\right)<\varepsilon \text{ and}\left|N_{\varepsilon }\left(p\right)\right|\geq MinPts\\ N_{\varepsilon }\left(p\right)=\left\{q\in P|d\left(p,q\right)<\varepsilon \right\} \end{array}\right. \end{array}$$ (8)

In Eq (8), $N_{\varepsilon }\left(P\right)$ is the point set in the neighborhood of point p; $\left|N_{\varepsilon }\left(p\right)\right|$ indicates the number of points in the neighborhood of a point. To further improve the clustering quality, the preliminary clustering results are post processed, including eliminating clusters with too few points and merging clusters with close spatial distance [26]. The final cluster merging decision needs to comprehensively consider the distance between clusters and the scale of clusters. The study is based on the merging criterion of relative distance to ensure the rationality and effectiveness of the merging operation, as shown in Eq (9).


$$\begin{array}{c} d\left(C_{i},C_{j}\right)<\gamma \cdot \left(diam\left(C_{i})+diam(C_{j}\right)\right) \end{array}$$ (9)

In Eq (9), $d\left(C_{i},C_{j}\right)$ is the distance between the center points of two clusters; $diam\left(C_{i}\right)$ and $diam\left(C_{j}\right)$ are the diameters of cluster $C_{i}$ and cluster $C_{j}$, respectively; γ is the merge threshold coefficient. γ controls inter cluster merging based on relative clustering diameter to avoid erroneous fusion of different objects. Finally, the label of each cluster and its BB are output to complete the task of PC segmentation. To rigorously handle sparsity and improve feature representation in 3D detection, the improved PointNet incorporates the following mathematical formulations. In complex dynamic scenes, the robustness of PC segmentation directly determines the stability of subsequent detection tasks. To improve the ability to suppress noise and outliers, the segmentation process is formalized as an optimization problem with regular constraints, and its calculation is shown in Eq (10).


$$\begin{array}{c} J_{\text{seg}}=\sum_{k=1}^{K}\sum_{p_{i}\in C_{k}}{\|p_{i}-\mu_{k}\|}^{\text{2}}+\lambda \sum_{k=1}^{K}\text{II}(\left|C_{k}\right|<\tau ) \end{array}$$ (10)

In Eq (10), $J_{\text{seg}}$ represents the segmentation objective function value, which measures the clustering quality. K represents the total number of clusters. $C_{k}$ represents the kth cluster. $p_{i}$ represents the ith point in the PC. $\mu_{k}$ represents the center point of the kth cluster. λ represents the regularization coefficient. II(·) represents the indicator function. τ represents the threshold for cluster size. Traditional fixed thresholds are difficult to adapt to the changing PC density distribution in road scenes. Therefore, the study introduces an adaptive distance threshold based on local statistics, as shown in Eq (11).


$$\begin{array}{c} \varepsilon \left(p\right)=a\cdot \frac{\text{1}}{\left|N_{r}\left(p\right)\right|}\sum_{q\in N_{r}\left(p\right)}\|p-q\| \end{array}$$ (11)

**Fig 2:**
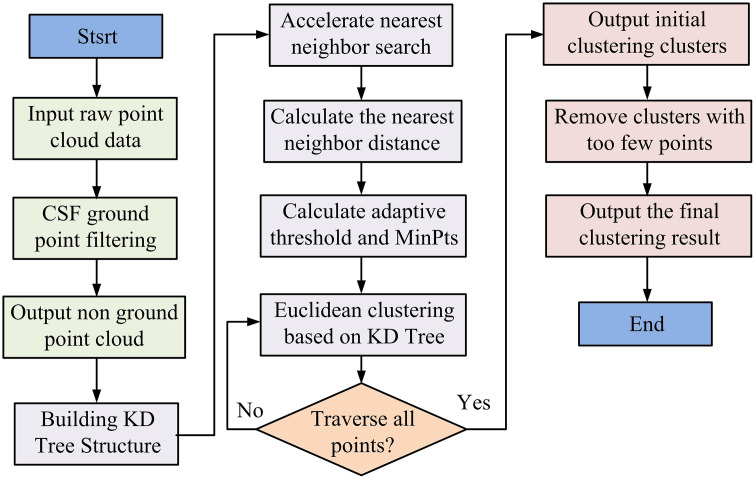
Flow diagram of improved EC algorithm.

In Eq (11), ε(p) represents the adaptive distance threshold at point p. a represents the scaling factor. $N_{r}\left(p\right)$ represents the set of neighboring points of point p within radius r. q represents a point in the neighborhood. The mathematical logic for clustering distance lies in introducing local statistics to enable the threshold to adaptively change with PC density. This essentially solves the problem of segmentation robustness under density changes by dynamically adjusting similarity measures. The above Eq (11) establishes a robustness model based on an adaptive threshold using local statistics, transforming the density change problem into local neighborhood statistical analysis, and achieving global optimization by adjusting the alpha parameter. The flow diagram of the improved EC algorithm is presented in Fig 2.

In Fig 2, the improved EC algorithm first performs CSF ground point filtering on the input raw PC to separate out non-ground PCs. Subsequently, a KD-Tree structure is constructed to accelerate neighbor search, and adaptive distance thresholds and minimum point counts are calculated. Based on these parameters, EC is performed, and after traversing all points, the initial clustering clusters are output. Finally, through post-processing steps such as eliminating too small clusters, the final high-quality clustering results are output.

### 2.2 3D OD method based on improved PointNet

After efficiently segmenting the PC, it is essential to further accurately identify and detect 3D objects from the segmented PC clusters. However, existing 3D OD methods exhibit poor real-time performance, and traditional PointNets tend to ignore detailed information when performing fine detection on complex 3D objects, leading to a decrease in detection accuracy [27]. Therefore, a 3D OD method based on an improved PointNet is proposed. This method extracts local features of 3D objects through MSG and MRG. To enhance the network’s feature integration capability, a skip connection mechanism is incorporated into the convolutional layer structure, the prediction of the affine transformation matrix is eliminated, and random point sampling is adopted to reduce computational complexity. The reason for removing the affine transformation matrix prediction module is that the T-Net structure requires an independent sub network for affine transformation prediction, which introduces additional computational overhead and parameter count [28].

The PointNet extracts features point by point through a Multi-Layer Perceptron (MLP) with shared weights and a symmetry function, and aggregates global information to achieve the detection task of 3D objects for vehicle-mounted lidar [29]. This network utilizes a Transform Network (T-Net) to align the coordinates of the input PC, reducing external transformation interference [30]. Then, multiple MLP layers with shared weights are independently applied to each point for feature extraction, gradually increasing the feature dimension of each point, and using max pooling as the symmetry function to aggregate the features of all points, as shown in Eq (12).


$$\begin{array}{c} \left\{ \begin{array}{c} @l@\overset{\left(l\right)}{\underset{i}{f}}=\text{ReLU(}W^{\left(l\right)}\cdot \overset{\left(l-1\right)}{\underset{i}{f}}+b^{\text{(}l\text{)}}\text{)}\\ G=\underset{i=1,\text{\textbackslash{}ldots},A}{max}\left(\overset{\left(L\right)}{\underset{i}{f}}\right) \end{array}\right. \end{array}$$ (12)

**Fig 3:**
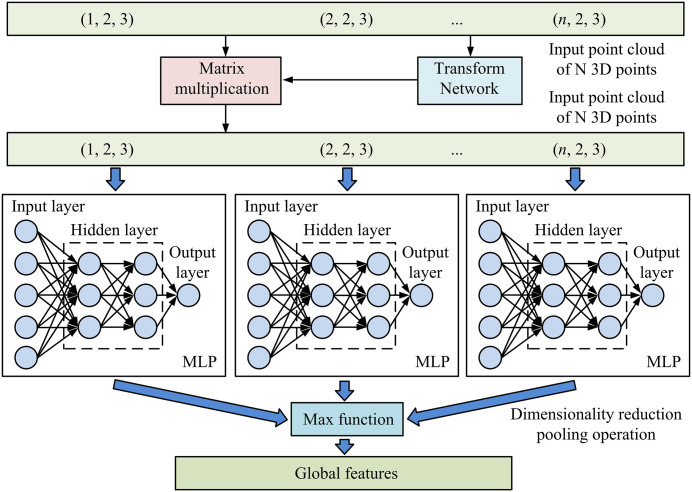
Algorithm architecture diagram of PointNet.

In Eq (12), $\overset{\left(l\right)}{\underset{i}{f}}$ is the output characteristics of the MLP at layer l; $W^{\left(l\right)}$ and $b^{\text{(}l\text{)}}$ are the weight matrix and bias vector of MLP; ReLU means activation function; G is the global eigenvector obtained after the maximum pool; A is the eigenvector of all points; L is the total number of layers of MLP. The algorithm architecture diagram of PointNet is shown in Fig 3.

Fig 3 shows the overall architecture of the PointNet algorithm. The network first performs spatial transformation alignment on N input 3D PCs to improve rotation invariance. Subsequently, high-dimensional features are extracted point by point using an MLP with shared weights, and the features of all points are aggregated using a max pooling function to generate a global feature vector. The entire process achieves end-to-end processing from raw PC input to global feature output, balancing point level feature learning and global information integration. To significantly improve processing efficiency while retaining key geometric information, the study first performs random point sampling on the input PC, uniformly downsampling it to a fixed number of points. The sampling process is presented in Eq (13).


$$\begin{array}{c} P_{\text{sampled}}=\text{RPS}\left(P_{\text{input}},M\right) \end{array}$$ (13)

In Eq (13), $P_{\text{sampled}}$ is the output PC after sampling; RPS means random point sampling function; $P_{\text{input}}$ indicates the input PC; M indicates the fixed number of points after sampling. The traditional PointNet lacks explicit modeling of local neighborhood structure. To overcome this defect and enable the network to perceive the local geometric context in different ranges, the study introduces an MSG strategy [31]. By querying the neighborhood of each point in multiple different radius ranges, the strategy can capture the local structural features at different scales from fine to coarse, as shown in Eq (14).


$$\begin{array}{c} \left\{ \begin{array}{c} @l@B\left(p_{i},r_{k}\right)=\left\{p_{j}|{\|p_{j}-p_{i}\|}_{\text{2}}<r_{k}\right\}\\ \overset{\left(k\right)}{\underset{local}{f}}=\underset{p_{j}\in B\left(p_{i},r_{k}\right)}{max}\left\{\text{MLP}\left(f_{j}\right)\right\} \end{array}\right. \end{array}$$ (14)

In Eq (14), $B\left(p_{i},r_{k}\right)$ is the neighborhood point set with point $p_{i}$ as the center and radius $r_{k}$ as; $p_{i}$ and $p_{j}$ are the center point and any other point in the PC respectively; $r_{k}$ is the search radius at the kth scale; *f*^(k)^_local_ is the local aggregation characteristics of point $p_{i}$ under scale k; $f_{j}$ is the input characteristics of the point $p_{j}$. The selection of MSG radius aims to capture hierarchical geometric features from fine-grained details to broader contextual structures. Capture local curvature and edges with a small radius (0.1 m). The medium radius (0.2 m) covers the component level structure. Large radius (0.4 m) covers object level context and partial occlusion. In addition, the density change of PC in different regions also serves as a crucial determinant influencing the effect of feature extraction. To enhance the adaptability of the network to sparse and dense areas, the study uses the MRG strategy to group the PCs, ensuring that the structural information can be effectively captured regardless of the point density [32], as shown in Eq (15).


$$\begin{array}{c} f_{i,mag}=\text{Concat}\overset{K}{\underset{k=1}{\left[\text{MLP}\left(\text{FPS}\left(P,s_{k}\right)\right)\right]}} \end{array}$$ (15)

In Eq (15), $f_{i,mag}$ is the multi-resolution aggregation feature; Concat indicates feature splicing operation; $s_{k}$ is the number of sampling points under scale k; $\text{FPS}\left(P,s_{k}\right)$ is the farthest point sampling function; $\overset{K}{\underset{k=1}{\left[\cdot \right]}}$ means traversing k different scales. To avoid gradient vanishing and information decay in deep networks, and to fully utilize geometric details in shallow layers and semantic information in deep layers, a skip connection mechanism is introduced. This mechanism directly fuses shallow and deep features through channel concatenation, forming composite feature vectors with richer information. The fusion method is presented in Eq (16).


$$\begin{array}{c} f_{\text{fused}}=\text{Concat}\left(f_{\text{global}},\overset{\left(1\right)}{\underset{\text{local}}{f}},\overset{\left(2\right)}{\underset{\text{local}}{f}},\text{\textbackslash{}ldots},\overset{\left(k\right)}{\underset{\text{local}}{f}}\right) \end{array}$$ (16)

In Eq (16), $f_{\text{fused}}$ is the composite feature vector after fusion; $f_{\text{global}}$ is the global eigenvector. Eq (16) establishes a sparse data feature fusion model through skip connections, transforming the information flow optimization problem into direct connections and concatenation operations in the feature space. These formulaic expressions not only define specific algorithm steps, but more importantly, transform abstract scientific goals such as robustness, efficiency, and sparse data processing capabilities into computable problems with clear objective functions, parameter spaces, and assessable indicators, thus establishing a solid bridge between theoretical rigor and practical effectiveness. Considering the uneven density of LiDAR PCs, a density weighted multi-resolution grouping strategy is proposed, as shown in Eq (17).


$$\begin{array}{c} {𝐟}_{\text{i,mrg}}=\text{Concat(}\rho_{b}\text{(}p_{i}\text{)}\cdot \text{MLP(FSP(}P,s_{b}\text{)))} \end{array}$$ (17)

In Eq (17), ${𝐟}_{\text{i,mrg}}$ represents the aggregated feature vector of point $p_{i}$ after density weighted multi-resolution grouping. $\rho_{b}\text{(}p_{i}\text{)}$ represents the local point density of point $p_{i}$ at the bth scale. $\text{FSP(}P,s_{b}\text{)}$ represents the farthest sampling function. P represents the collection of the entire input PC. $s_{b}$ represents the number of sampling points at the bth scale. In the detection header part, the network outputs the category probability and BB parameters of each candidate target. The boundary box regression needs to precisely forecast the location and dimensions of the target. The smooth L1 loss function can effectively balance the advantages of the loss and provide a more stable gradient at the initial stage of training [33]. The expression is presented in Eq (18).


$$\begin{array}{c} L_{\text{reg}}=\sum_{e}{\text{smooth}}_{L\text{1}}(b_{e}-{\hat{b}}_{e}) \end{array}$$ (18)

In Eq (18), $L_{\text{reg}}$ is the smooth L1 loss function; e refers to sample index; $b_{e}$ and ${\hat{b}}_{e}$ are the predicted BB parameters and the real BB parameters of the eth target, respectively. Category prediction uses cross entropy loss, which can accurately assess the discrepancy between the predicted probability distribution and the actual label, and promote the network to learn the correct category discrimination characteristics [34]. Finally, through the weighted combination of regression loss and classification loss, the network can simultaneously optimize the target positioning and classification performance, and realize end-to-end training, as shown in Eq (19).


$$\begin{array}{c} \left\{ \begin{array}{c} @l@L_{\text{cls}}=-\sum_{c}y_{c}\;log\;\left({\hat{y}}_{c}\right)\\ L^{\prime }=L_{\text{reg}}+\lambda L_{\text{cls}} \end{array}\right. \end{array}$$ (19)

**Fig 4:**
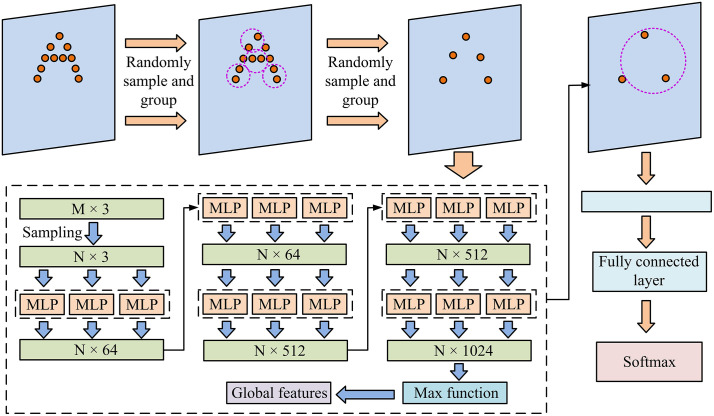
Schematic diagram of improved PointNet.

In Eq (19), $L_{\text{cls}}$ is classification loss; c indicates category index; $y_{c}$ and ${\hat{y}}_{c}$ are the category probability of the real label and prediction respectively; $L^{\prime }$ is the total loss function; λ indicates the balance superparameter. For the loss function, its mathematical logic is reflected in balancing classification and localization losses, so that the model can maintain detection accuracy while satisfying real-time constraints. The illustrative schematic of the improved PointNet is presented in Fig 4.

**Fig 5:**
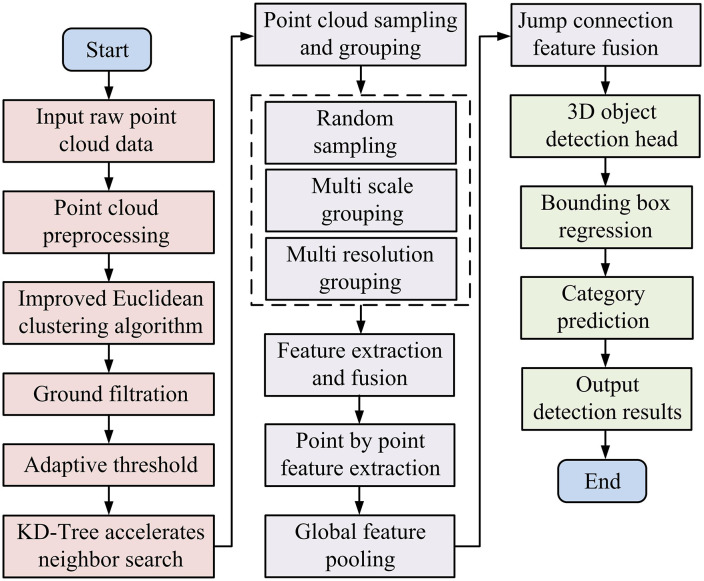
The flowchart of 3D object detection using vehicle mounted LiDAR.

In Fig 4, the improved PointNet first performs random sampling and grouping on the input PC, and then extracts high-dimensional features layer by layer through a series of MLPs with shared weights. The network aggregates global features using the max pooling function and integrates multi-level information through skip connections, ultimately outputting detection results through a fully connected layer and a Softmax classifier. The flowchart of 3D object detection using vehicle mounted LiDAR is shown in Fig 5.

In Fig 5, firstly, the original point cloud data is input, and after preprocessing, an improved Euclidean clustering algorithm is used for ground filtering and adaptive threshold clustering, and KD Tree is used to accelerate neighborhood search. Then perform random sampling, multi-scale grouping, and multi-resolution grouping on the point cloud. Subsequently, feature extraction and fusion are carried out through point by point feature extraction and global feature pooling, and a skip connection mechanism is introduced. Finally, the 3D object detection head completes bounding box regression and category prediction, and outputs the detection results. The entire process has achieved end-to-end processing from point cloud input to object detection.

## 3 Results

The PC segmentation capability of the introduced improved EC algorithm was evaluated to verify its resilience and instantaneous processing capability in complex scenes. Subsequently, the 3D OD performance of the improved PointNet was verified, aiming to assess its detection accuracy and efficiency under multi-category and multi-difficulty objects.

### 3.1 Evaluation of PC segmentation performance for improved EC algorithm

To verify the performance of the proposed improved EC algorithm in the task of PC segmentation for vehicle-mounted LiDAR, a systematic evaluation was conducted on two public datasets: KITTI and NuScenes. The KITTI dataset contains PC data from urban road scenes, with annotations covering categories such as vehicles, pedestrians, and bicycles. The NuScenes dataset includes PC data under more complex multi-scene and multi-weather conditions. The experimental evaluation was conducted using publicly available datasets, ensuring reproducibility and comparability. For the KITTI dataset, data were collected with a vehicle-mounted Velodyne HDL-64E LiDAR, which has a maximum range of ~120 m, a 360° horizontal field of view, and 64 laser channels. The NuScenes dataset employs a 32-beam LiDAR with comparable horizontal coverage and an effective detection range of up to 100 m for object-level perception.

Four representative clustering algorithms were selected as benchmark comparisons. Traditional EC serves as a baseline reference using fixed distance thresholds [14]. Fast Channel Clustering (FCC) represents the hardware-accelerated approach for extreme speed optimization [10]. Mask-based Panoptic LiDAR Segmentation (MaskPLS) embodies advanced segmentation methods incorporating semantic information [11]. The Real-Time Point Cloud Clustering algorithm for Roadside LiDAR (RTPCC-RL) demonstrates application-specific optimization [12]. By systematically comparing with these four types of methods covering different technical routes, the comprehensive advantages of the proposed improved algorithm in accuracy, speed, and adaptability were fully validated. The experimental environment and parameter settings are shown in Table 1.

**Table 1:** Experimental setup and parameter configurations.

Experimental environment		Parameter settings	
Central processing unit	Intel Xeon Gold 6248R @ 3.0GHz	Learning rate	0.001
Graphics processing unit	NVIDIA RTX 3090 (24GB)	Batch size	16
Memory	128 GB DDR4	Weight decay	0.01
Operating system	Ubuntu 20.04 LTS	Iterations	600
Programming language	Python 3.8	Cluster radius	1.2
DL framework	PyTorch 1.13.1 + CUDA 11.7	Minimum points MinPts	5

To verify the rationality of the keyword parameter settings in the proposed algorithm, sensitivity analysis was conducted on the coefficients α, β, and γ on the KITTI and NuScenes datasets, and the Intersection over Union (IoU) and processing time of the proposed algorithm were evaluated. α, β, and γ were mainly determined through iterative grid search process, and their selection was based on achieving the best balance between segmentation accuracy and computational efficiency. The purpose of this search is to find parameter values that can generalize well under different scene complexities and point cloud densities inherent in these benchmark datasets. The sensitivity analysis results are shown in Table 2. In Table 2, in the KITTI dataset, when the coefficient α is set to 1.2, the IoU reaches the highest value of 92.12% with a processing time of 15.63 ms. On the NuScenes dataset, the optimal IoU is also obtained when α is set to 1.2, which is 90.89% with a processing time of 17.24 ms. For the coefficient β, when it is set to 0.5, the optimal IoU is obtained on both datasets with corresponding processing times of 15.63 ms and 17.24 ms. The coefficient γ performs best when it is set to 0.8, with IoUs of 92.12% and 90.89% on the KITTI and NuScenes datasets, respectively. Comprehensive analysis shows that α is 1.2, β is 0.5, and γ is 0.8, which can achieve low processing delay while maintaining high segmentation accuracy, verifying the effectiveness and robustness of this parameter setting on different datasets.

**Table 2:** Sensitivity analysis of coefficients α, β, and γ on two datasets.

Coefficients	Tested values	KITTI		NuScenes	
		IoU (%)	Processing Time (ms)	IoU (%)	Processing Time (ms)
α	0.8	90.12	14.95	88.45	16.88
	1.0	91.78	15.30	90.12	17.02
	1.2	92.12	15.63	90.89	17.24
	1.4	91.05	16.20	89.67	17.55
β	0.3	89.45	14050	88.20	16.95
	0.4	91.20	15.10	89.85	17.10
	0.5	92.12	15.63	90.89	17.24
	0.6	91.55	16.40	90.12	17.65
γ	0.6	91.80	15.20	89.95	17.15
	0.7	91.95	15.50	90.32	17.20
	0.8	92.12	15.63	90.89	17.24
	0.9	91.30	15.90	89.78	17.35

**Fig 6:**
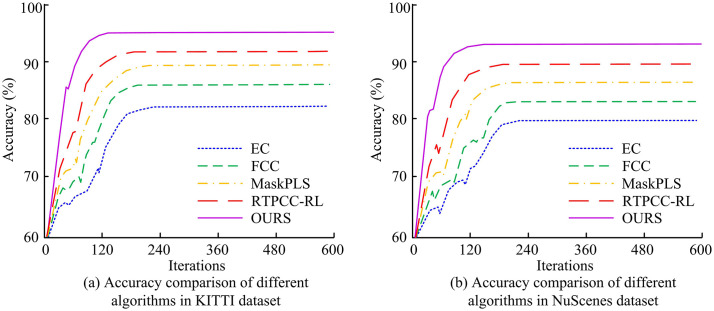
The accuracy of various algorithms in the KITTI and NuScenes datasets.

The study first analyzed the segmentation accuracy of the proposed improved EC algorithm on PCs in the KITTI and NuScenes datasets, and compared it with other mainstream clustering algorithms. The outcomes are presented in Fig 6. In Fig 6(a), in the KITTI dataset, when the iteration count was 120, the accuracies of EC, FCC, MaskPLS, and RTPCC-RL algorithms were 71.63%, 78.76%, 85.09%, and 89.31%, respectively, while the accuracy of the introduced algorithm was 94.26%. When the iteration count reached 240, the accuracies of EC, FCC, MaskPLS, RTPCC-RL, and the introduced algorithm were 81.76%, 85.23%, 88.39%, 91.26%, and 94.96%. In Fig 6(b), when the iteration count was 120, the accuracies of the five clustering algorithms in the NuScenes dataset were 69.89%, 76.02%, 80.05%, 87.92%, and 92.24%. When the iteration count increased to 240, the accuracies of EC, FCC, MaskPLS, and RTPCC-RL algorithms were 79.27%, 82.86%, 86.37%, and 89.12%, respectively, while the accuracy of the introduced algorithm was 93.12%. The outcomes showed that the introduced algorithm had superior segmentation accuracy and robustness in both datasets.

**Fig 7:**
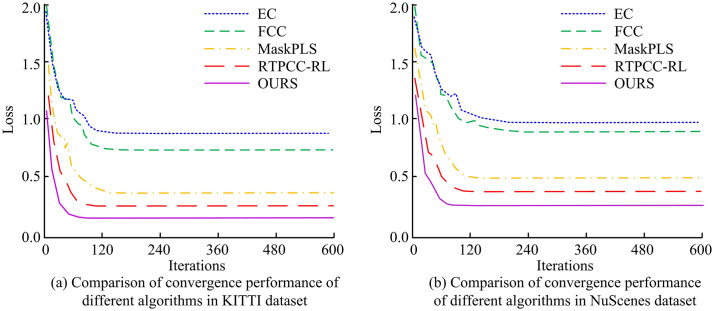
The loss values of different algorithms in KITTI and NuScenes datasets.

To confirm the convergence performance of the introduced algorithm, a comparison of the losses of different clustering algorithms was carried out on the KITTI and NuScenes datasets, with the results shown in Fig 7. In Fig 7(a), on the KITTI dataset, at an iteration count of 60, the losses of EC, FCC, and MaskPLS algorithms were 1.22, 1.77, and 0.74, while the losses of RTPCC-RL and the introduced algorithm were 0.38 and 0.23, respectively. When the iteration count reached 200, the losses of EC, FCC, MaskPLS, RTPCC-RL, and the introduced algorithm were 0.89, 0.77, 0.38, 0.28, and 0.20, respectively. In Fig 7(b), on the NuScenes dataset, at an iteration count of 60, the losses of the five algorithms were 1.26, 1.23, 0.86, 0.54, and 0.38, respectively. When the iteration count increased to 200, the losses of EC, FCC, MaskPLS, and RTPCC-RL algorithms were 0.97, 0.88, 0.48, and 0.37. Compared to these, the loss of the introduced algorithm was 0.26, representing reductions of 73.20%, 70.79%, 46.94%, and 31.58%. The outcomes indicated that the introduced algorithm exhibited superior convergence and stability in both datasets, effectively enhancing clustering efficiency.

The study further analyzed the comprehensive performance of different clustering algorithms on two datasets, with assessment criteria encompassing precision, recall, F1 score, and IoU. The results are presented in Table 3. In Table 3, the precision, recall, and F1 score of the introduced algorithm on the KITTI dataset were 0.943, 0.935, and 0.939, respectively, with an IoU of 92.12%, all of which were superior to other comparative algorithms. On the NuScenes dataset, the precision, recall, F1 score, and IoU of the introduced algorithm were 0.928, 0.914, 0.921, and 90.89%, respectively. Specifically, the precision of the introduced algorithm was 10.02%, 8.9%, 4.95%, and 2.16% higher than that of EC, FCC, MaskPLS, and RTPCC-RL algorithms, respectively, and the IoU was 14.47%, 12.26%, 8.04%, and 3.68% higher, respectively. The outcomes demonstrated that the introduced algorithm could effectively balance the accuracy and completeness of segmentation, achieving more precise and robust PC segmentation effects in different scenarios.

**Table 3:** Performance comparison of various clustering algorithms.

Datasets	Algorithms	Precision	Recall	F1 score	IoU (%)
KITTI	EC	0.852	0.821	0.836	78.35
	FCC	0.872	0.839	0.855	80.50
	MaskPLS	0.904	0.882	0.893	84.92
	RTPCC-RL	0.917	0.898	0.907	88.73
	The proposed algorithm	0.943	0.935	0.939	92.12
NuScenes	EC	0.835	0.802	0.818	76.42
	FCC	0.852	0.821	0.836	78.63
	MaskPLS	0.890	0.865	0.877	82.85
	RTPCC-RL	0.908	0.890	0.899	87.21
	The proposed algorithm	0.928	0.914	0.921	90.89

**Fig 8:**
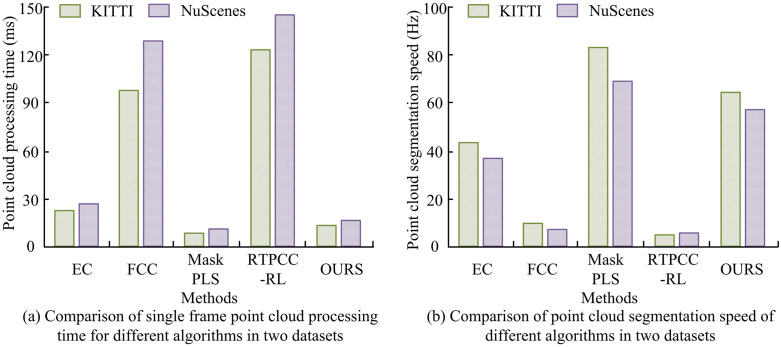
Single frame PC processing time and PC segmentation speed of different algorithms in KITTI and NuScenes datasets.

To confirm the efficacy of the introduced algorithm, the study compared the processing time of single-frame PCs and the speed of PC segmentation among different algorithms on two datasets. The outcomes are presented in Fig 8. In Fig 8(a), the processing times of single-frame PCs for the EC, FCC, MaskPLS, and RTPCC-RL algorithms on the KITTI dataset were 22.52ms, 98.36ms, 12.34ms, and 120.56ms, respectively, while on the NuScenes dataset, they were 25.84ms, 135.72ms, 14.85ms, and 147.92ms, respectively. The processing times of the introduced algorithm on the two datasets were 15.63ms and 17.24ms, respectively. In Fig 8(b), on the KITTI dataset, the PC segmentation speeds of EC, FCC, MaskPLS, RTPCC-RL, and the introduced algorithm were 44.42 Hz, 10.26Hz, 82.04Hz, 4.96Hz, and 67.13 Hz, respectively. The segmentation speeds of these five algorithms on the NuScenes dataset were 38.82 Hz, 7.43 Hz, 67.68 Hz, 5.36 Hz, and 58.06 Hz. The outcomes demonstrated that the introduced algorithm could effectively improve computational efficiency while ensuring segmentation accuracy. Compared with FCC and RTPCC-RL algorithms, the processing time of single frames and the speed of PC segmentation were improved by nearly 10 times, meeting the real-time PC processing requirements of auto drive systems.

### 3.2 Verification of improved 3D OD performance of PointNet

To confirm the capability of the proposed improved PointNet for 3D OD, the study analyzed its detection accuracy and efficiency on two datasets, KITTI and NuScenes, and compared it with existing popular 3D OD methods. The comparative methods encompassed mainstream 3D detection technical approaches: traditional PointNet served as the baseline method for direct PC processing [29]. PointNet++ served as a representative PC network for hierarchical feature extraction [30]. PointPillars represented the efficient pillar-based encoding detection approach [35]. Voxel RCNN reflected the balanced design of voxel-based methods [36]. PV-RCNN stood as the current performance benchmark for point-voxel fusion frameworks [37]. Through systematic comparison with these methods based on different design philosophies, the significant improvements of the improved PointNet in detection accuracy, inference speed, and overall performance were conclusively demonstrated.

The study first conducted sensitivity analysis on the MSG radius in the KITTI and NuScenes datasets, using mAP and NuScenes Detection Score (NDS) as evaluation metrics. The results are shown in Table 4. In Table 4, the radius combinations (0.1, 0.2, 0.4) achieved optimal results on both the KITTI and NuScenes datasets, with mAP of 90.36% and NDS of 81.07% on KITTI. On NuScenes, mAP was 89.03% and NDS was 80.15%. The results indicated that the selected combination achieved the best balance between capturing local details and contextual information, and had strong cross dataset generalization ability. The selected radius corresponded to a typical physical scale in urban driving scenarios, with a radius of 0.1m being the width of pedestrian limbs or vehicle edges. A radius of 0.2m was half the width of a compact car door. A radius of 0.3m was a typical distance between objects in congested traffic. This geometric alignment ensured that the features learned by the network were semantically meaningful and could be extended across object categories.

**Table 4:** Sensitivity analysis of MSG radii on detection performance.

Radius Set (r_1_, r_2_, r_3)_ (m)	KITTI		NuScenes	
	mAP (%)	NDS (%)	mAP (%)	NDS (%)
(0.05, 0.1, 0.2)	85.23	73.48	84.12	72.35
(0.1, 0.2, 0.3)	87.91	77.25	86.45	76.82
(0.1, 0.2, 0.4)	90.36	81.07	89.03	80.15
(0.2, 0.3, 0.5)	88.74	79.33	87.68	78.92
(0.2, 0.4, 0.6)	86.52	76.81	85.79	75.43

**Fig 9:**
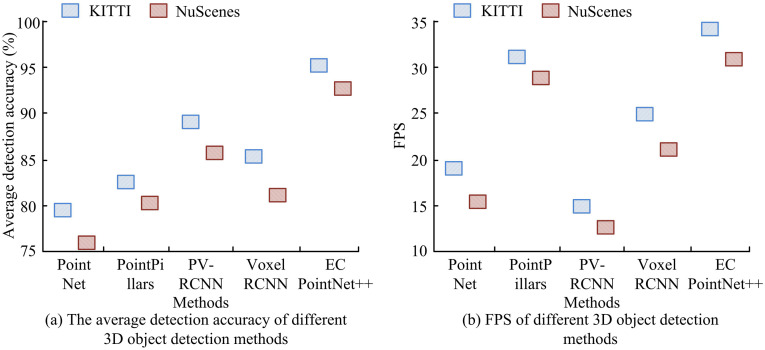
The average detection accuracy and FPS of different 3D OD methods.

The study compared the average detection accuracy and average Frames Per Second (FPS) of different 3D OD methods on the KITTI and NuScenes datasets, aiming to investigate the detection accuracy and instantaneous processing capability of the introduced approach. The outcomes are presented in Fig 9. In Fig 9(a), the average detection accuracy of PointNet, PointPillars, and PointRCNN on the KITTI dataset was 79.65%, 82.47%, and 88.58%, while the average detection accuracy of sparse depth-based single shot detector and the proposed method EC PointNet++ was 85.78% and 94.36%, respectively. On the NuScenes dataset, the average detection accuracy of PointNet, PointPillars, PV-RCNN, and Voxel RCNN was 75.84%, 80.05%, 85.97%, and 81.26%, while the accuracy of the proposed method EC PointNet++ was 92.68%. In Fig 9(b), the FPS of PointNet, PointPillars, PV-RCNN, Voxel RCNN, and the introduced approach on the KITTI dataset were 19fps, 31fps, 14fps, 24fps, and 34fps, respectively, while on the NuScenes dataset, they were 16fps, 23fps, 12fps, 21fps, and 31fps, respectively. The outcomes indicated that the proposed 3D OD method achieved high detection precision while enhancing processing speed, achieving a balance between accuracy and speed.

**Fig 10:**
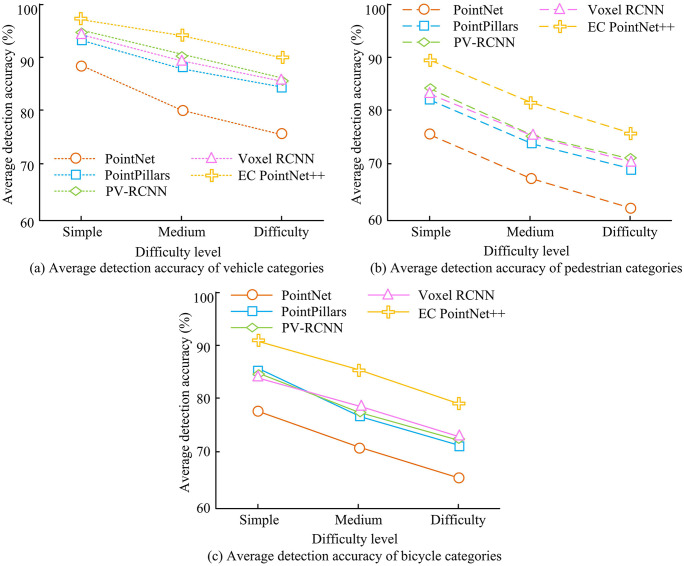
The average detection accuracy of three types of targets using different 3D OD methods.

To confirm the detection capability of the proposed detection method under different categories and difficulty levels, the study analyzed the average detection accuracy of different detection methods for three types of targets: vehicles, pedestrians, and bicycles, in the KITTI dataset. The difficulty levels were categorized as easy, medium, and hard. The outcomes are presented in Fig 10. In Fig 10(a), the detection accuracy of PointNet, PointPillars, PV-RCNN, Voxel RCNN, and the introduced approach for vehicles under the easy level were 88.92%, 92.13%, 94.80%, 94.24%, and 97.21%, while the accuracy under the hard level were 76.35%, 85.24%, 86.78%, 86.52%, and 90.89%. In Fig 10(b), for pedestrian detection, the detection accuracy of the five methods under the easy level were 75.68%, 82.23%, 83.81%, 82.86%, and 89.24%, respectively, while the accuracy under the hard level were 62.13%, 68.37%, 73.26%, 72.89%, and 76.61%, respectively. In Fig 10(c), for bicycle detection, the detection accuracy of PointNet, PointPillars, PV-RCNN, and Voxel RCNN under the hard level were 65.13%, 72.01%, 73.04%, and 74.09%, respectively, while the detection accuracy of the proposed method EC PointNet++ was 79.06%. The outcomes indicated that the introduced approach could effectively enhance the feature representation ability for multi-category and multi-difficulty targets, achieving superior detection accuracy and improving the 3D detection performance in complex scenes.

The study conducted ablation experiments on the proposed 3D OD method in two datasets, with evaluation metrics including accuracy and training time. The results are shown in Table 5. In Table 5, the combination of modules produced significant synergistic effects. The combination of MSG and MRG resulted in mAPs of 85.10% and 82.95% on the KITTI and NuScenes datasets, respectively. The mAP of MSG+Skip and MRG+Skip, which contain skip connections, further increased to 86.33 and 86.89% on the KITTI dataset, and 84.12 and 84.67% on the NuScenes dataset, respectively. Introducing only a dynamic distance threshold could increase the mAP of the KITTI dataset from 75.52% to 80.15%, and the mAP of the NuScenes dataset from 73.81% to 78.40%, with only a slight increase in training time. This confirmed that adaptive thresholding based on local PC density could significantly improve segmentation accuracy, especially in areas where PC density changes. The logarithmic point adaptive mechanism further increased the mAP of KITTI dataset to 81.33% and NuScenes dataset to 79.88%. This indicated its role in balancing clustering sensitivity and computational efficiency, effectively preventing over segmentation in dense PCs and under segmentation in sparse PCs. Only adding skip connections could increase the mAP of KITTI dataset to 83.44% and NuScenes dataset to 81.28%. In theory, skip connections alleviated the problem of gradient vanishing and promote better gradient flow during backpropagation. More importantly, they achieved direct feature reuse by concatenating shallow geometric details with deep semantic features. This multi-level feature fusion enhanced the model’s ability to simultaneously represent fine-grained local structures and high-level contextual information, thereby improving detection accuracy and stability, especially in complex and occluded scenes. The full model achieved the best detection accuracy, with mAPs of 88.96% and 86.27% on KITTI and NuScenes, respectively, which were 13.44 and 12.46 percentage points higher than the baseline model. It is worth noting that the training time for the complete model was 12.32 minutes and 12.81 minutes respectively, indicating that collaborative optimization between modules improved training efficiency.

**Table 5:** Ablation study of the proposed improved PointNet on KITTI and NuScenes datasets.

Model Variant	KITTI		NuScenes	
	mAP (%)	Training time/ epoch (min)	mAP (%)	Training time/ epoch (min)
Baseline	75.52	11.96	73.81	12.47
+ Dynamic Distance Threshold	80.15	12.30	78.40	12.85
+ Logarithmic Point Adaptation	81.33	12.15	79.88	12.70
+ Affine Transformation Matrix	79.85	14.20	77.92	14.85
+ RPS only	78.20	13.02	76.45	13.62
+ MSG only	80.26	13.15	78.19	14.03
+ MRG only	81.67	13.58	79.63	14.35
+ Skip Connections only	83.44	12.87	81.28	13.88
+ Dynamic Distance Threshold+ Logarithmic Point Adaptation	83.90	12.60	82.15	13.20
+ MSG + MRG	85.10	14.58	82.95	15.20
+ MSG + Skip	86.33	14.21	84.12	14.85
+ MRG + Skip	86.89	14.45	84.67	15.10
Full model	88.96	12.32	86.27	12.81

To explore the actual detection performance of the introduced approach, a comparison was carried out regarding the average detection accuracy and detection speed of different 3D OD methods in real-world urban road scenes. The testing of traffic flow is conducted in a high-density mixed traffic flow simulation scenario. This scenario covers various dynamic traffic participants, such as passenger cars, buses, and pedestrians, and includes complex interactive scenarios such as intersection queuing and lane changing games. In this test, the traffic flow was set at approximately 2500–3200 vehicles per hour, with passenger cars accounting for about 75%, buses accounting for about 10%, and other participants such as pedestrians and cyclists accounting for about 15%. Data collection is conducted under various lighting conditions, including sunny, cloudy, and nighttime. To focus on the perceptual stability of the model under standard operating conditions, all tests were conducted without precipitation or extreme weather events. The research and testing routes cover various structured types of urban roads, including main roads, secondary roads, and intersections with traffic lights. The real-world test data was collected using a vehicle platform on selected urban roads in Nanjing, Jiangsu Province, China. The test vehicle is equipped with Velodyne HDL-64E LiDAR, which is consistent with the main sensor of KITTI benchmark test, to ensure the fairness of point cloud feature comparison. The collected dataset consists of continuous LiDAR point cloud frames that record the defined urban scenes. At the same time, corresponding high-precision GPS/INS data was recorded as trajectory truth values, and manual 3D bounding box annotation was performed on the main targets (vehicles, pedestrians, cyclists) in some keyframes for quantitative evaluation. Due to the current stage of the project and institutional data policies, this specific real-world dataset has not yet been publicly released. However, the evaluation process and detailed annotation specifications (including definitions of target category, occlusion level, truncation degree, and bounding box specifications) follow the established standards of the publicly available KITTI 3D object detection benchmark.

**Fig 11:**
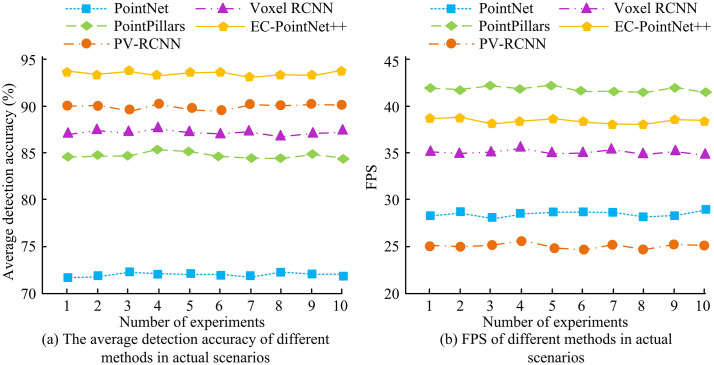
Average detection accuracy and speed of different methods in practical scenarios.

The outcomes are presented in Fig 11. In Fig 11(a), the average detection accuracies of PointNet, PointPillars, and PV-RCNN in actual urban road scenes were 72.31%, 84.57%, and 90.01%, respectively. The average detection accuracies of Voxel RCNN and the introduced approach were 87.21% and 93.86%. Compared with the other four approaches, the detection accuracy of the introduced approach was improved by 21.55%, 9.29%, 3.84%, and 6.64%, respectively. In Fig 11(b), the average FPS of PointNet, PointPillars, PV-RCNN, Voxel RCNN, and the introduced approach were 28fps, 42fps, 25fps, 35fps, and 38fps, respectively. The outcomes indicated that the proposed 3D OD method exhibited superior detection accuracy and efficiency in real-world scenes, outperforming other comparative methods and validating its superiority.

In Table 6, the study further analyzed the detection performance of the proposed method on the KITTI dataset at different difficulty levels. It should be noted that the detection difficulty of the KITTI dataset was divided into three levels based on the degree of target occlusion, truncation, and bounding box size: Easy, Moderate, and Hard. This grading standard provided an important benchmark for evaluating the robustness of the model in different challenging scenarios. The proposed method significantly outperformed mainstream methods in terms of overall performance and difficulty levels. Specifically, the mAP of the proposed method was as high as 91.04%, which was 16.64%, 13.19%, 9.34%, and 6.59% higher than PointNet, PointNet++, PointPillars, Voxel RCNN, and PV-RCNN, respectively. In simple, moderate, and difficult difficulty scenarios, the AP values of the proposed method were 94.36%, 91.24%, and 87.52%, respectively, which were significantly ahead of other methods, especially in difficult scenarios. The results fully demonstrated the effectiveness of the proposed method in improving detection accuracy and scene robustness.

**Table 6:** Performance comparison of different 3D OD methods on the KITTI dataset.

Detection method	mAP (%)	Easy AP (%)	Moderate AP (%)	Hard AP (%)	*p*-value
PointNet	74.40	79.65	75.21	68.34	<0.001
PointNet++	78.15	83.20	78.90	72.10	<0.001
PointPillars	77.85	82.47	78.93	72.15	<0.001
Voxel RCNN	81.70	85.78	82.45	76.88	<0.001
PV-RCNN	84.45	88.58	85.12	79.64	<0.01
EC PointNet++	91.04	94.36	91.24	87.52	/

This study compared and analyzed the average accuracy, Average Translation Error (ATE), Average Scale Error (ASE), and NDS of different 3D OD methods on the NuScenes dataset. The results are shown in Table 7. In Table 7, the proposed method achieved optimal results in all key indicators. Specifically, the average accuracy mAP of the proposed method was 92.68%, significantly higher than PointNet, PointNet++, PointPillars, Voxel RCNN, and PV-RCNN. In terms of positioning accuracy, the proposed method had ATE and ASE as low as 0.298m and 0.218m, respectively, which were superior to all compared methods. In the end, the NDS of the proposed method reached 83.45%, which was significantly improved compared to other methods. These data fully demonstrated the excellent detection performance and accuracy advantages of the proposed method in complex multi scene scenarios.

**Table 7:** Comprehensive performance comparison of different 3D OD methods on the NuScenes dataset.

Detection method	mAP (%)	ATE (m)	ASE (m)	NDS (%)	*p*-value
PointNet	75.84	0.412	0.281	68.35	<0.001
PointNet++	79.20	0.398	0.270	71.85	<0.001
PointPillars	80.05	0.385	0.264	72.14	<0.001
Voxel RCNN	85.78	0.351	0.243	76.89	<0.001
PV-RCNN	88.58	0.328	0.231	79.62	<0.01
EC PointNet++	92.68	0.298	0.218	83.45	/

To evaluate the robustness of the proposed method in challenging environments, the sparsity of PCs was simulated by randomly downsampling the original LiDAR frames to 10%, 20%, and 50% of their original points under sparse PC conditions. The mAP and FPS of different methods were compared and analyzed, and the results are shown in Table 8. In Table 8, the proposed method achieved mAP of 84.37% and 82.15% on the KITTI and NuScenes datasets, respectively, which was over 16 percentage points higher than PointNet. When the density increased to 50%, the mAP of the proposed method reached 91.85% and 90.03%, respectively, maintaining a leading advantage. In terms of real-time performance, the proposed method maintained a stable frame rate of 31 FPS-33 FPS, slightly lower than PointPillars’ maximum of 38 FPS, but achieved a better balance between accuracy and speed. The results indicated that the proposed method significantly enhances the robustness to sparse PCs through improved clustering strategies and feature extraction mechanisms.

**Table 8:** Performance under sparse PC conditions.

PC density	Methods	KITTI mAP (%)	*p*-value	NuScenes mAP (%)	*p*-value	FPS
10%	PointNet	68.21	<0.001	65.43	<0.001	22
	PointNet++	75.89	<0.001	73.20	<0.001	19
	PointPillars	78.34	<0.001	76.55	<0.001	35
	EC-PointNet++	84.37	/	82.15	/	31
20%	PointNet	72.18	<0.001	70.11	<0.001	23
	PointNet++	79.45	<0.001	77.33	<0.001	20
	PointPillars	81.26	<0.001	79.87	<0.001	36
	EC-PointNet++	88.92	/	86.44	/	32
50%	PointNet	76.33	<0.001	74.68	<0.001	24
	PointNet++	83.12	<0.001	81.05	<0.001	21
	PointPillars	84.77	<0.001	83.22	<0.001	38
	EC-PointNet++	91.85	/	90.03	/	33

Further comparative analysis was conducted on the mAP, Precision, Recall, F1 score, and FPS of different methods in real-world urban driving scenarios. The results are shown in Table 9. In Table 9, the proposed method ranked first with a mAP of 91.42%, which was 1.3 percentage points higher than the second place PV-RCNN. In terms of quality indicators, the proposed method achieved the best performance with an accuracy of 0.928, a recall of 0.912, and an F1 score of 0.920. In terms of real-time performance, the proposed method significantly outperformed PV-RCNN and PointNet++ with a processing speed of 36 FPS, second only to PointPillars which focused on speed optimization. These data proved that the method in this paper could maintain high precision detection and good real-time performance at the same time in complex real scenes, and meet the actual deployment requirements of auto drive system.

**Table 9:** Performance in real-world urban driving scenarios.

Methods	mAP (%)	*p*-value	Precision	Recall	F1-Score	FPS
PointNet	78.63	<0.001	0.802	0.776	0.789	26
PointNet++	85.20	<0.001	0.868	0.842	0.855	22
PointPillars	87.91	<0.001	0.894	0.870	0.882	40
Voxel RCNN	88.47	<0.001	0.901	0.878	0.889	32
PV-RCNN	90.12	<0.001	0.917	0.896	0.906	28
EC-PointNet++	91.42	/	0.928	0.912	0.920	36

**Fig 12:**
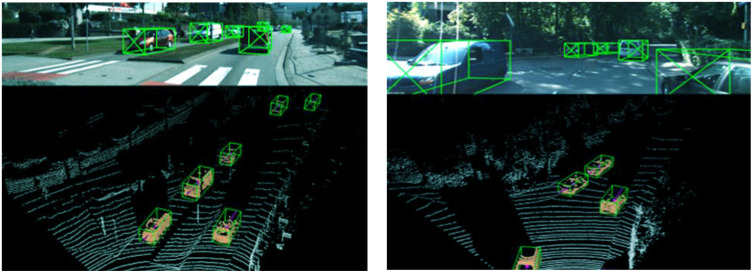
Improving the 3D OD performance of PointNet network. (Taken in Nanjing, Jiangsu, China, October 2024).

To further investigate the actual detection performance of the proposed improved PointNet network for 3D objects, the study conducted tests in a real urban scene, and the results are shown in Fig 12. In Fig 12, it was found that the improved PointNet network could achieve stable and accurate detection of vehicles, and the generated 3D bounding boxes were highly consistent with the true shape of the target. In complex environments with dense targets and partial occlusion, the improved network still exhibited strong robustness, reducing missed and false detections. The results indicated that the proposed method could achieve more complete and accurate 3D OD in complex environments, improving the robustness and practicality of the model.

## 4. Discussion

Addressing the shortcomings of traditional vehicle-mounted LiDAR 3D OD methods, which suffer from poor accuracy and low efficiency, this study proposed a novel method based on an improved EC algorithm and an enhanced PointNet. This method was validated on the KITTI and NuScenes datasets. Experimental findings demonstrated that the proposed improved EC algorithm achieved segmentation accuracies of 94.96% and 93.12% on the KITTI and NuScenes datasets, respectively, with single-frame processing times of 15.63 ms and 17.24 ms. These results significantly outperformed traditional methods such as standard EC, which achieved 81.76% accuracy at 22.52 ms on KITTI. The performance gain was attributed to the integrated preprocessing pipeline: the CSF ground filter effectively removed terrain interference, while the KD-Tree structure and the adaptive parameter mechanism enhanced robustness against density variations. Compared to the specialized FPGA-based fast clustering method [13], the proposed approach offers superior versatility and accuracy without hardware dependencies. Furthermore, compared to the roadside LiDAR clustering algorithm [15], the proposed method demonstrates a significant advance in real-time capability, being approximately 5.8 times faster, while also improving the segmentation F1 score by 3.2% on the NuScenes dataset. This balance between high accuracy and computational efficiency meets the stringent real-time requirements of AD perception systems.

In the 3D OD task, the proposed enhanced PointNet achieved average detection accuracies of 94.36% and 92.68% on KITTI and NuScenes, respectively, outperforming PointNet (79.65%), PointPillars (82.47%), and PV-RCNN (88.58%) on KITTI. The detection speed reached 34 FPS and 31 FPS, effectively balancing latency and precision. The ablation study confirmed the contribution of each component: while the baseline model achieved 75.52% mAP on KITTI, the integration of MSG, MRG, and skip connections progressively improved performance, with the full model reaching 88.96% mAP. The MSG strategy with radii (0.1, 0.2, 0.4)m proved optimal, yielding an mAP of 90.36% and NDS of 81.07% on KITTI, as it captured multi-scale features from fine edges to object-level context. For challenging targets, the method maintained robust performance, achieving 90.89%, 76.61%, and 79.06% AP for hard-level vehicles, pedestrians, and cyclists on KITTI, respectively. This demonstrated enhanced feature representation for occluded and sparse objects. Moreover, under simulated sparse conditions (10% point density), the model retained an mAP of 84.37% on KITTI, showcasing significantly better robustness compared to PointNet (68.21%). The method’s practical efficacy was validated in real urban scenes, attaining 93.86% detection accuracy at 38 FPS. These comprehensive results validated that the synergistic integration of improved clustering for precise proposal generation and enhanced DL for discriminative feature extraction effectively addressed the core challenges in vehicle-mounted LiDAR 3D OD.

## 5. Conclusion

This study presented a 3D OD method based on an improved EC algorithm and an enhanced PointNet for vehicle-mounted LiDAR systems. The contributions of the research were threefold. First, a PC segmentation framework was developed by integrating Cloth Simulation Filtering, KD-Tree neighbor search, and an adaptive parameter mechanism, which improved both the accuracy and efficiency of clustering under varying point densities. Second, an enhanced PointNet architecture was designed by incorporating multi-scale grouping, multi-resolution grouping, and skip connections, leading to more robust feature extraction and fusion. Third, the proposed method was validated on the KITTI and NuScenes datasets, where it achieved superior segmentation accuracy, detection performance, and real-time processing capability compared to existing approaches.

Several directions for future research are recommended. First, self-supervised or weakly-supervised pre-training strategies should be explored to enhance model generalization, particularly in extremely sparse or noisy PC scenarios. Second, the integration of complementary sensor modalities, such as thermal cameras or radar, could be investigated to improve robustness under adverse weather and lighting conditions. Third, the computational efficiency of the detection network should be further optimized through lightweight network design or model compression techniques to facilitate deployment on embedded platforms. Finally, extensive real-world testing in diverse and unstructured driving environments is needed to validate the practical applicability and safety of the proposed system.

## Supporting information

S1 File Minimal data set definition. (DOC)
