# Supplementary material for: 3D object detection for vehicle-mounted LiDAR based on deep learning and euclidean clustering algorithm
Source: PLoS One. 2026 Jun 1;21(6):e0348581. doi: 10.1371/journal.pone.0348581 (PMC13225636; doi:10.1371/journal.pone.0348581)
Supplement: S1 File — (DOC) [file pone.0348581.s001.doc]

**The data in Figure 5**

| Datasets | Iterations | Accuracy (%) | | | | |
| --- | --- | --- | --- | --- | --- | --- |
|  |  | EC | FCC | MaskPLS | RTRCC-RL | OURS |
| KITTI | 120 | 71.63 | 78.74 | 85.07 | 89.29 | 94.26 |
|  | 240 | 81.76 | 85.21 | 88.37 | 91.24 | 94.96 |
|  | 360 | 81.76 | 85.21 | 88.37 | 91.24 | 94.96 |
|  | 480 | 81.76 | 85.21 | 88.37 | 91.24 | 94.96 |
|  | 600 | 81.76 | 85.21 | 88.37 | 91.24 | 94.96 |
| NuScenes | 120 | 69.89 | 76.02 | 80.05 | 87.92 | 92.24 |
|  | 240 | 79.27 | 82.84 | 86.35 | 89.10 | 93.12 |
|  | 360 | 79.27 | 82.84 | 86.35 | 89.10 | 93.12 |
|  | 480 | 79.27 | 82.84 | 86.35 | 89.10 | 93.12 |
|  | 600 | 79.27 | 82.84 | 86.35 | 89.10 | 93.12 |

**The data in Figure 6**

| Datasets | Iterations | Loss | | | | |
| --- | --- | --- | --- | --- | --- | --- |
|  |  | EC | FCC | MaskPLS | RTRCC-RL | OURS |
| KITTI | 120 | 0.92 | 0.79 | 0.38 | 0.28 | 0.20 |
|  | 240 | 0.89 | 0.77 | 0.38 | 0.28 | 0.20 |
|  | 360 | 0.89 | 0.77 | 0.38 | 0.28 | 0.20 |
|  | 480 | 0.89 | 0.77 | 0.38 | 0.28 | 0.20 |
|  | 600 | 0.89 | 0.77 | 0.38 | 0.28 | 0.20 |
| NuScenes | 120 | 1.07 | 0.95 | 0.50 | 0.38 | 0.26 |
|  | 240 | 0.97 | 0.89 | 0.49 | 0.38 | 0.26 |
|  | 360 | 0.97 | 0.89 | 0.49 | 0.38 | 0.26 |
|  | 480 | 0.97 | 0.89 | 0.49 | 0.38 | 0.26 |
|  | 600 | 0.97 | 0.89 | 0.49 | 0.38 | 0.26 |

**The data in Figure 7**

| Methods | KITTI | | NuScenes | |
| --- | --- | --- | --- | --- |
|  | Point cloud processing time (ms) | Point cloud segmentation speed (Hz) | Point cloud processing time (ms) | Point cloud segmentation speed (Hz) |
| EC | 22.52 | 44.42 | 25.84 | 38.82 |
| FCC | 92.37 | 10.25 | 135.72 | 7.43 |
| MaskPLS | 12.35 | 82.05 | 14.85 | 67.68 |
| RTRCC-RL | 120.57 | 4.97 | 147.92 | 5.36 |
| OURS | 15.63 | 67.13 | 17.24 | 58.06 |

**The data in Figure 8**

| Methods | KITTI | | NuScenes | |
| --- | --- | --- | --- | --- |
|  | Average detection accuracy (%) | FPS | Average detection accuracy (%) | FPS |
| PointNet | 79.65 | 19 | 75.84 | 16 |
| PointPillars | 82.47 | 31 | 80.05 | 23 |
| PV-RCNN | 88.58 | 14 | 85.98 | 12 |
| Voxel RCNN | 85.78 | 24 | 81.27 | 21 |
| OURS | 94.36 | 34 | 92.68 | 31 |

**The data in Figure 9**

| Target detection category | Methods | Average detection accuracy (%) | | |
| --- | --- | --- | --- | --- |
|  |  | Simple | Medium | Difficulty |
| Vehicle | PointNet | 88.92 | 80.05 | 76.35 |
|  | PointPillars | 92.13 | 89.02 | 85.24 |
|  | PV-RCNN | 94.81 | 89.76 | 86.78 |
|  | Voxel RCNN | 94.25 | 90.27 | 86.52 |
|  | OURS | 97.21 | 94.13 | 90.89 |
| Pedestrian | PointNet | 75.68 | 67.25 | 62.13 |
|  | PointPillars | 82.23 | 74.66 | 68.37 |
|  | PV-RCNN | 83.81 | 76.87 | 73.26 |
|  | Voxel RCNN | 82.86 | 76.58 | 72.89 |
|  | OURS | 89.24 | 81.59 | 76.61 |
| Bicycle | PointNet | 78.32 | 71.27 | 65.13 |
|  | PointPillars | 85.15 | 77.58 | 72.01 |
|  | PV-RCNN | 84.71 | 78.36 | 73.05 |
|  | Voxel RCNN | 83.89 | 79.10 | 74.10 |
|  | OURS | 90.69 | 86.92 | 79.06 |

**The data in Figure 10 (a)**

| Number of experiments | Average detection accuracy (%) | | | | |
| --- | --- | --- | --- | --- | --- |
|  | PointNet | PointPillars | PV-RCNN | Voxel RCNN | OURS |
| 1 | 71.81 | 84.18 | 89.02 | 86.21 | 93.88 |
| 2 | 71.85 | 84.26 | 89.09 | 87.46 | 93.71 |
| 3 | 72.77 | 84.26 | 89.57 | 86.80 | 94.25 |
| 4 | 72.46 | 85.17 | 90.52 | 87.34 | 93.77 |
| 5 | 72.47 | 85.09 | 89.96 | 86.35 | 94.18 |
| 6 | 71.97 | 84.49 | 89.86 | 87.51 | 94.15 |
| 7 | 71.83 | 84.42 | 90.48 | 87.75 | 93.32 |
| 8 | 72.67 | 84.45 | 90.54 | 87.20 | 93.57 |
| 9 | 72.59 | 84.93 | 90.59 | 87.53 | 93.62 |
| 10 | 72.68 | 84.45 | 90.57 | 88.05 | 94.15 |

**The data in Figure 10 (b)**

| Number of experiments | FPS | | | | |
| --- | --- | --- | --- | --- | --- |
|  | PointNet | PointPillars | PV-RCNN | Voxel RCNN | OURS |
| 1 | 28 | 42 | 25 | 35 | 38 |
| 2 | 29 | 42 | 25 | 35 | 38 |
| 3 | 27 | 43 | 25 | 35 | 37 |
| 4 | 28 | 42 | 26 | 36 | 37 |
| 5 | 28 | 43 | 24 | 35 | 38 |
| 6 | 28 | 42 | 24 | 35 | 37 |
| 7 | 28 | 41 | 25 | 36 | 37 |
| 8 | 27 | 41 | 24 | 34 | 37 |
| 9 | 27 | 42 | 25 | 35 | 39 |
| 10 | 28 | 41 | 25 | 34 | 38 |
